# Evaluation of Aggregate Oral Fluid Sampling for Early Detection of African Swine Fever Virus Infection

**DOI:** 10.3390/v17081089

**Published:** 2025-08-06

**Authors:** Bonto Faburay, Kathleen O’Hara, Marta Remmenga, Theophilus Odoom, Sherry Johnson, William Tasiame, Matilda Ayim-Akonor, Benita Anderson, Kingsley Kwabena Amoako, Diane Holder, Wu Ping, Michelle Zajac, Vivian O’Donnell, Lizhe Xu, Robin Holland, Corrie Brown, Randall Levings, Suelee Robbe-Austerman

**Affiliations:** 1 Foreign Animal Disease Diagnostic Laboratory, National Bio and Agro-Defense Facility, Animal and Plant Health Inspection Service, United States Department of Agriculture, Manhattan, KS 66502, USA; diane.j.holder@usda.gov (D.H.); michelle.zajac@usda.gov (M.Z.); robin.holland@usda.gov (R.H.); 2 Center for Epidemiology and Animal Health, Strategy and Policy, Animal and Plant Health Inspection Service, Veterinary Services, United States Department of Agriculture, Fort Collins, CO 80523, USA; 3 Veterinary Services Department, Ministry of Food and Agriculture, Accra M-37, Ghana; theodoom@yahoo.com (T.O.); btdjagmah@yahoo.com (B.A.); kamoako15@gmail.com (K.K.A.); 4 School of Veterinary Medicine, University of Ghana, Accra LG-25, Ghana; sajohnson@ug.edu.gh; 5 School of Veterinary Medicine, Kwame Nkrumah University of Science and Technology, Kumasi 00233, Ghana; drwilly2002@gmail.com; 6 CSIR-Animal Research Institute, Accra P.O. Box AH 20, Ghana; m.ayimakonor@gmail.com; 7 Foreign Animal Disease Diagnostic Laboratory (FADDL), National Veterinary Services Laboratories, Animal and Plant Health Inspection Service, United State Department of Agriculture, Plum Island Animal Disease Center, Plum Island, Orient, NY 11957, USA; ping.wu@usda.gov (W.P.); vivian.odonnell@usda.gov (V.O.); lizhe.xu@usda.gov (L.X.); 8 LifeStock International, Athens, GA 30606, USA; corrie@lifestock.org; 9 National Veterinary Services Laboratories, Animal and Plant Health Inspection Service, Veterinary Services, United States Department of Agriculture, Ames, IA 50010, USA; Randall.Levings@usda.gov (R.L.); suelee.robbe-austerman@usda.gov (S.R.-A.)

**Keywords:** African swine fever, African swine fever virus, surveillance, aggregate oral fluid, blood, oropharyngeal swabs

## Abstract

African swine fever (ASF) needs to be controlled, and prevention of the spread of African swine fever virus (ASFV) is dependent on enhanced surveillance and early disease detection. Commercial swine operations, especially in North America, Europe, and Asia, are characterized by comparatively large numbers of pigs, and sampling individual pigs, which represents the main strategy for current ASF surveillance, can be both costly and labor intensive. A study performed in Ghana was designed to estimate the diagnostic sensitivity of pen-based aggregate oral fluid testing for ASFV in infected pigs in a pen of 30 animals and to evaluate its utility as a tool to support surveillance of ASF in the US. This study was performed in three phases: (i) virus (Ghana ASFV24) amplification in a target host species to generate the challenge inoculum; (ii) titration of the inoculum (10% spleen homogenate) in target host species to determine the minimum dose inducing acute ASF in pigs with survival up to 5–6 days post-inoculation (dpi); and (iii) the main study, involving 186 pigs, consisting of 6 replicates of 30 pigs per pen and one seeder pig inoculated with wildtype ASFV (highly virulent genotype II) per pen. Daily sampling of aggregate oral fluids, uncoagulated blood, oropharyngeal swabs, fecal and water nipple swabs, and recording of rectal temperatures and clinical observations was carried out. The seeder pigs were each inoculated intramuscularly with 0.5 mL of the 10% spleen homogenate, which induced the desired clinical course of ASF in the pigs, with survival of up to 6 dpi. ASFV DNA was detected in the seeder pigs as early as 1 dpi and 2 dpi in the blood and oropharyngeal swabs, respectively. Transmission of ASFV from the seeder pigs to the contact pig population was detected via positive amplification of ASFV DNA in aggregate oral fluid samples at 3 days post-contact (dpc) in 4 out of 6 pens, and in all 6 pens, at 4 dpc. Testing of oropharyngeal swabs and blood samples from individual pigs revealed a variable number of ASFV-positive pigs between 3 and 5 dpc, with detection of 100% positivity between 6 and 18 dpc, the study endpoint. These findings demonstrate the potential utility of aggregate oral fluid sampling for sensitive and early detection of ASFV incursion into naïve swine herds. It also demonstrates that testing of environmental samples from the premises could further enhance overall ASF early detection and surveillance strategies.

## 1. Introduction

African swine fever (ASF) is a highly contagious transboundary viral hemorrhagic disease of domestic and wild pigs presenting a significant threat to the global swine industry [1,2,3,4,5,6,7]. Consequently, ASF is listed as reportable by the World Organization for Animal Health (WOAH) [8]. It is caused by African swine fever virus (ASFV), a large double-stranded DNA virus and the sole member of the family *Asfaviridae* and the genus *Asfivirus*. It is a highly complex virus with a genome size that ranges from 170 to 190 kbp, encoding 150–200 proteins, including 68 structural proteins and more than 100 non-structural proteins, depending on the virus isolate [9,10].

Originally, the emergence and outbreak of ASFV outside Africa was caused by genotype I; however, genotype II is now responsible for most of the outbreaks globally [3,11,12,13]. Following its introduction in the Caucasus, Georgia, in 2007, the p72 genotype II of ASFV has since spread globally, causing disease outbreaks in Russia (2007), the Baltic States and Eastern Europe (2014), Belgium (2018), China (2018), Vietnam (2019), Cambodia (2019), North Korea (2019), Germany (2020), and most recently, in the Dominican Republic (July 2021) [14,15,16,17]. The recent outbreak of ASF in the Caribbean, specifically in the Dominican Republic and Haiti, presents a significant risk of introduction onto US mainland with devastating economic consequences, including restrictions on international trade in swine, pork, and pork products. Except for Vietnam where two vaccines have been recently approved for controlled use in the field [18,19,20,21,22], there are currently no effective and widely available vaccines, and, therefore, control and prevention of spread of the virus are dependent on enhanced surveillance and early detection. Enhancing preparedness for early virus detection requires implementation of robust surveillance using sample types and sampling methods that allow for sensitive and timely detection of ASFV. Commercial swine operations, especially in North America, Europe, and Asia, are characterized by a comparatively large number of pigs, and sampling individual pigs for blood, the approved sample type by the USDA and WOAH, which represents the main strategy for current ASF surveillance, is costly, labor intensive, and unsustainable.

Oral fluid (OF) is a complex matrix containing an assortment of proteins, including antibodies, mucin, and a variety of enteric microorganisms as well as metabolites recovered by pigs from their environment [23,24]. Thus, as a result of pigs’ normal exploratory behavior (smelling, tasting, and biting, etc.), environmental diagnostic targets are collected in the buccal cavity [25], which may subsequently be passed onto the rope and into the oral fluid. This makes oral fluid a suitable sample type for use to support surveillance of a variety of swine pathogens [26,27]. Oral fluid testing is based on the use of a non-invasive aggregate sampling used in industry and several laboratories in the US for diagnostic testing and surveillance for a variety of endemic swine pathogens, such as swine enteric coronavirus diseases [28,29], porcine reproductive and respiratory syndrome [30], swine influenza A virus [31], and other viruses [32,33].

Importantly, oral fluid has been shown to be an acceptable sample type for detection of ASFV [12,34,35]. The primary route transmission of ASFV in pigs is via ingestion; the tonsils and oropharynx represent the sites of initial virus replication [36,37]. Consequently, ASFV may be detected early in the pharyngeal swabs and oral fluid [38] prior to the onset of clinical signs in infected pigs. A recent study concluded that following introduction of ASFV into the southeastern part of the United States, a rapid response could prevent up to 79% of virus spread in the region [39]. Additionally, validation and subsequent adoption of aggregate oral fluids as an approved sample type by industry in North America and the WOAH for ASFV early detection and surveillance will significantly reduce cost of surveillance, enhance disease control, and improve profitability of the swine industry. However, studies exploring aggregate OF samples for ASFV detection have left gaps in understanding sample performance in situations of low disease prevalence. Additionally, consistencies in sampling individual pigs have made conclusive interpretations and inferences challenging. The aim of this study was to estimate the diagnostic sensitivity of pen-based aggregate OF testing for ASFV in infected pigs in a pen of 30 animals and evaluate its utility as a tool to support surveillance for ASF in the United States and North America generally.

## 2. Materials and Methods

### 2.1. Ethics Statement

This study was conducted in accordance with the highest ethical standards. All experimental procedures were approved by the University of Ghana Institutional Animal Care and Use Committee with the Approval Code UG-IACUC048/23-24 and approval date of 23 April 2024.

### 2.2. Production of ASFV Inoculum

#### 2.2.1. The Virus and Preparation of Inoculum

A virulent ASFV isolate, obtained from an ASF outbreak in a smallholder pig farm in Ghana (now named Ghana ASFV24), was used for the experimental inoculation of the pigs. The virus has been shown to be highly lethal, causing 100% mortality in the affected farm. To prepare the virus inoculum for the study, a spleen tissue obtained previously from a pig that died from ASF was used. Approximately 1 g of the tissue was transferred into a sterile mortar, with the addition of 0.5g of sterile sand, and ground manually using a sterile pestle. Thereafter, 9 mL of sterile phosphate buffered saline (PBS) was added, and the mixture further homogenized to produce a 10% tissue homogenate. Aliquots of the homogenate were transferred into 2 mL sterile microcentrifuge tubes and centrifuged at 2000 rpm for 5 min. Supernatants were transferred into clean 2 mL tubes and stored at −80 °C until used.

#### 2.2.2. Virus Amplification and Determination of Transmissibility

An experiment was carried out to amplify the challenge inoculum as well as assess the virulence and transmissibility of the challenge virus. Two naïve pigs weighing approximately 20 kg each were selected for the inoculation. One pig (pig A) was inoculated intramuscularly with 2 mL of the ASFV 10% spleen homogenate. The second naïve pig (pig B) was left uninoculated and housed with the inoculated pig to facilitate virus transmission. The animals were monitored daily for onset and progression of clinical signs, including pyrexia (≥40 °C), with daily collection and testing of whole blood. Blood samples were tested for viremia daily by quantitative PCR (qPCR) based on the protocol used at the National Veterinary Services Laboratories’ Foreign Animal Disease Diagnostic Laboratory (FADDL) [38], as described below. At a Ct value of less than 18, the inoculated pig (A) was euthanized, and spleen samples were harvested, and aliquots of the 10% homogenate were prepared as described above. Monitoring of the transmission and disease progression continued in pig B until it succumbed to ASF at 11 dpc.

#### 2.2.3. Whole-Genome Sequencing and Phylogenetic Analysis

To determine genotype classification and further characterize the genome of Ghana ASFV24 in future studies, the ASFV inoculum sequence was analyzed by whole-genome sequencing on an Oxford Nanopore Technologies (ONT., Oxfordshire, Oxford, UK) GridION sequencing platform with the ONT Rapid Barcoding kit (RBK114.96) and on Illumina MiSeq 2000 (Ilumina Inc., San Diego, CA, USA) with the Nextera XT library preparation kit and using NextSeq 2000 P3 Reagents (300 cycles) (Ilumina Inc., San Diego, CA, USA). In addition, the sample was amplified with a PCR-tiled primer set developed by Warr et al. [40] and with a p72-specific primer set [41]. The amplicons, after purification using Ampure XP beads (Beckman Coulter Life Science, Indianapolis, IN, USA), were sequenced on the ONT platform. The combined data from the different NGS runs were assembled with CLC Genomics Workbench (Qiagen, Hilden, Germany) version 24.0.2, using the De Novo Assemble Long Reads and Polish with Short Reads workflow. After analyzing the contigs with the NCBI blastn tool to identify the closest isolate from the GenBank on the *B646L* gene, the data was assembled again using the isolate as a reference in CLC. Multiple sequence alignment of the generated *B646L* sequence with sequences retrieved from GenBank was performed in CLC Genomics Workbench. A Maximum Likelihood phylogenetic tree was constructed in CLC with 1000 bootstraps or iterations.

### 2.3. In Vivo Dose Titration

To determine the minimal dose that induces acute ASF in seeder pigs with survival of up to 5–6 dpi, three different doses of the 10% spleen homogenate, low (0.5 mL), medium (1 mL), and high (2 mL), were evaluated in 3 groups of pigs consisting of 3 animals per group. The animals were inoculated via the intramuscular route, and daily whole blood samples in EDTA were collected from each animal. DNA was extracted as described below. Viral load was measured by ASFV qPCR according to the protocol described below. The animals were monitored daily for clinical signs. Clinical signs were scored according to the parameters described previously, with modifications [42,43] (Table S1), and a clinical score of 4 or above or death served as an endpoint. The study ended on day 8 post-inoculation.

### 2.4. Isolation and Quantification of Virus Inoculum

Primary swine blood macrophages were prepared from defibrinated swine blood, as described elsewhere [44]. Briefly, heparin-treated swine blood was incubated at 37 °C for 1–2 h to allow sedimentation of the erythrocyte fraction. Mononuclear leukocytes were separated by a Ficoll-Paque (Pharmacia, Piscataway, NJ, USA) density gradient (specific gravity, 1.079). The monocyte/macrophage cell fraction was cultured in Primaria T-75 tissue culture flasks (Corning Inc., Corning, NY, USA) containing complete macrophage media, composed of the RPMI 1640 medium (Life Technologies), with 30% L929 supernatant, 20% heat-inactivated—gamma-irradiated fetal bovine serum (Sigma, St. Louis, MO, USA), 1 X antibiotics/antimycotics, 1 X Gentamicin (Thermo Fisher Scientific (Gibco), Waltham, MA, USA), and complementary plasma for 24 h at 37 °C under 5% CO_2_. Adherent cells were detached from the Primaria flasks by using 10 mM of EDTA in Dulbeco’s phosphate buffered saline (DPBS, Thermo Fisher Scientific) and were then reseeded into Primaria 6-well plates or 96-well plates and incubated at 37 °C in 5% CO_2_ for use in assays 24 h later. Virus titration was performed on primary macrophages in Primaria 96-well plates. Briefly, 10-fold serial dilutions of the prepared sample (10% homogenate) were prepared in duplicate in complete macrophage media. A volume of 50 uL/well per dilution (4 wells per dilution), in duplicate, were added to the Primaria 96-well tissue culture plates and seeded at a density of 5 × 10^6^ cells/plate. The plate was incubated at 37 °C in 5% CO_2_. The macrophages were observed daily under a microscope for up to 10 days, and detection of ASFV was determined by hemadsorption (HA). Virus concentrations are reported as 50% hemadsorption dose per ml (HAD_50_/mL), calculated by the Reed and Muench method [45].

### 2.5. Main Study

#### 2.5.1. Animals

**Figure 1:**
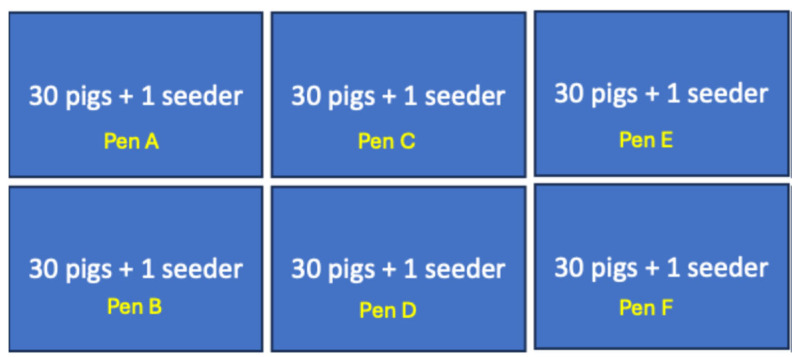
Layout of the study pens consisting of six replicates of 30 pigs and 1 seeder pig per pen. The location of the different pens (Pen A–F) is shown.

A total of 186 pigs, weighing 25–30 kg each, were purchased from a government swine-breeding facility in Accra, Ghana. The facility had no reported history of recent ASF cases or outbreaks. To enroll experimental animals into the study, serum samples were collected randomly from a subset of pigs (*n* = 96) from the farm to rule out prior exposure to ASFV via detection of antibodies using a validated commercial kit, according to manufacturer’s instructions (IDVet Indirect antibody ELISA, Innovative Diagnostics, Montpellier, France). The experiment to evaluate aggregate oral fluids was performed in an animal facility located at the Accra Veterinary Laboratory, Directorate of Veterinary Services, and consisted of 6 pen replicates, with each pen holding 30 pigs and one seeder pig (Figure 1). Water was provided ad libitum, and the animals were fed twice daily.

#### 2.5.2. Inoculation of Seeder Pigs and Contact Transmission

Following acclimation and rope training, one pig from each of the 6 groups was randomly selected as a “seeder pig” and inoculated intramuscularly with 0.5 mL of the 10% spleen homogenate of the wildtype ASFV described above. Selection of the seeder pigs was performed through a random count of 31 pigs in each pen, and selection of the tenth count as the seeder pig per pen or herd. Baseline aggregate oral fluids from each pen, EDTA blood, and oropharyngeal swabs from individual contact pigs were collected. Each inoculated seeder pig was introduced immediately into their respective pens to co-mingle with the contact pigs to facilitate contact transmission. This timepoint was considered 0 dpi for seeder pigs and 0 dpc for contact pigs.

### 2.6. Sampling and Clinical Data Collection

#### 2.6.1. Aggregate Oral Fluids

Aggregate oral fluid samples were collected from contact pigs in each of the pens daily (0–8 dpc) and on alternate days (10–22 dpc). After a week of acclimation, the pigs were subjected to an initial pre-study conditioning to rope chewing for 1 h. During each aggregate oral fluid collection, a single cotton rope (IDEXX Laboratories, Westbrook, ME, USA) was hung in each pen on a metal crossbar at shoulder-height level of the pigs. Contact pigs were allowed to chew on the rope for 30 min, with monitoring of individual pig-chewing activity and documentation of the proportion of pigs that chewed on the rope on any given day. The rope from each pen was collected, and oral fluid was squeezed out into a tube and transported to the laboratory for further processing. During aggregate oral fluid collection, seeder pigs were isolated in specifically designed pens to prevent them from chewing on the rope and were released back into their respective pen population after completion of each sampling to facilitate contact transmission. Isolation of the seeder pigs was necessary to avoid confounding effects of ASFV DNA detection in rope samples due to potential seeder pig contamination and to unequivocally demonstrate that ASFV DNA detection in aggregate oral fluids was the result of ASFV infection and transmission in contact pigs with oral shedding of the virus.

#### 2.6.2. Blood and Oropharyngeal Swabs

Uncoagulated blood samples in ethylenediaminetetraacetic acid (EDTA) in vacutainer tubes were collected daily from individual pigs from day 0 to day 8 dpc and on alternate days until 22 dpc, the study endpoint. The samples were temporarily stored at −80 °C. Prior to use, the samples were centrifuged at 3000 rpm for 30 min, and the supernatant was collected for DNA extraction, as described above. Oropharyngeal blood swabs were also taken daily using individual sterile polyester-tipped swabs in 1 mL of viral transport medium (VTM) (Teknova, Hollister, CA, USA) daily from individual pigs from 0 to 8 dpc and on alternate days, 10–22 dpc, until the study endpoint. The samples were stored at −80 °C before processing or processed for DNA extraction immediately.

#### 2.6.3. Environmental Samples: Fecal and Water Nipple Swabs

To evaluate potential utility of environmental sampling to support ASF surveillance, composite fecal swabs were collected daily from the middle and the four corners of each pen using sterile polyester-tipped swabs in 1 mL of sterile PBS from 0 to 8 dpc. Similarly, composite water nipple swabs were also taken from each pen using individual sterile polyester-tipped swabs in 1 mL of VTM from 0 to 10 dpc. These samples were stored at −80 °C until processing for DNA extraction.

#### 2.6.4. Clinical Data

Rectal temperatures and clinical scores [42,46] of individual pigs, based on previously described criteria (Table S1), were recorded daily until the end of the study at 22 dpc. In cases where death did not occur overnight, pigs demonstrating a clinical score equal to or greater than 4 were euthanized. Necropsies were performed on a subset of pigs to collect representative samples and ascertain the cause of death.

### 2.7. DNA Extraction and Quantitative Real-Time PCR (qPCR)

DNA extraction from aggregate oral fluids, whole blood, oropharyngeal swabs, and water nipple and fecal swabs was performed using the Qiagen DNA extraction kit according to manufacturer’s instructions, with modifications, using 100 µL of the sample added to 120 µL of lysis buffer, 20 µL of proteinase K, and 3 µL of the internal control Xeno. The DNA was eluted in 50 µL of elution buffer.

ASFV genomic DNA was detected using quantitative real-time PCR, targeting the p72 open reading frame [38]. Beta actin was used as the internal extraction and amplification control [47]. The qPCR reaction was carried out as described previously [34] in a reaction mix containing the Taqman Fast 1-step master mix (Thermo Fisher Scientific, Waltham, MA, USA) and was amplified using the Bio-Rad CFX96 instrument (Bio-Rad Laboratories Canada Ltd., Mississauga, ON, Canada) in 45 amplification cycles (50 °C for 5 min, 95 °C for 20 s, 95 °C for 3 s, and 60 °C for 30 s).

### 2.8. Data Analysis

For comparing sensitivity of ASFV DNA detection in aggregate oral fluids with detection in individual blood and oropharyngeal swabs collected from contact pigs, we computed the proportion of pens and pigs (for blood and oropharyngeal swabs) that was positive at each timepoint post-contact. For blood and oropharyngeal swabs, due to the large variations in the percentage of positive animals per timepoint, we calculated the median positivity for each timepoint. To analyze the trend in Ct values (viral load) for contact pigs across pens, we computed mean pen Ct values with standard deviations at each timepoint. These values were plotted to demonstrate the trend over time in the Ct value changes, indicative of changes in viral load. The Kaplan–Meier survival estimate was used to analyze time to death and survival rates for the dose titration study. Data was analyzed using GraphPad Prism 10.

## 3. Results

### 3.1. Production and Amplification of Virus Inoculum

Inoculation of Pig A with the 10% spleen homogenate with the virulent wildtype ASFV resulted in acute ASF, exhibiting characteristic clinical signs, including an elevated rectal temperature, cutaneous cyanosis, depression, and lethargy. Upon necropsy, the animal exhibited typical lesions for ASF gross pathological changes and lesions such as splenomegaly, hemorrhage, and lymphadenopathy. The animal developed detectable viremia, as determined by qPCR, at 1 dpi, with a progressive increase in viral load, as demonstrated by a reduction in cycle threshold (Ct) values from Ct 35 at 1 dpi to Ct 15 at 4 dpi. The pig was euthanized at 4 dpi, and the spleen was harvested and stored at −80 °C and later used to prepare 10% homogenate for use as a challenge inoculum in the in vivo dose titration and in subsequent experiments. Pig B, which was co-housed with Pig A, developed detectable viremia at 8 dpc as well as clinical symptoms characteristic of acute ASF, thus confirming transmissibility of the virus from infected to naïve hosts. Pig B died from ASF at 11 dpc.

### 3.2. In Vivo Dose Titration

To determine the minimum dose that would induce acute ASF in the seeder pigs with survival of up to 5–6 dpi, three different doses of the 10% spleen homogenate, low (0.5 mL), medium (1 mL), and high (2 mL), were evaluated, as described above, in 3 groups of pigs, Group A, B, and C, respectively. In vitro quantification of the virus inoculum in porcine primary macrophages indicated a titer of 4.43 HAD_50_/mL.

**Figure 2:**
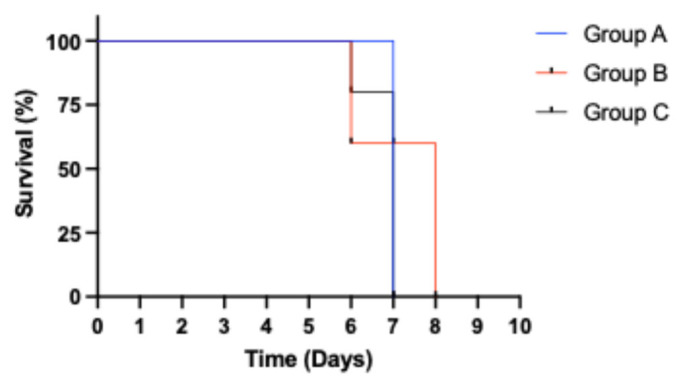
Kaplan–Meier survival analysis showing time of survival for animals in the different dose groups. Only pigs in Group A (0.5 mL) survived to 6 dpi without a mortality event.

Animals in all three groups exhibited elevated temperatures and developed viremia at 1 dpi (Ct value range: 22.04–33.34). There was a progressive reduction in Ct values with increasing severity clinical symptoms among animals in all three groups, Group A (Ct value range: 14.1–16.1), Group B (Ct value range: 15.96–16.71), and Group C (Ct value range: 14.97–15.8). Comparing responses among the different groups, animals that received the 0.5 mL homogenate (4.43 HAD_50_/mL) in Group A exhibited the longest survival time of 6 days without a mortality event (Figure 2). Consequently, this dose was used to inoculate the seeder pigs in the aggregate oral fluid evaluation study.

### 3.3. Whole-Genome Sequencing and Phylogenetic Analysis A

Whole-genome sequencing of ASFV inoculum revealed a genome size of approximately 185 kbp, which was within the size-range of ASFV genomes (170–190 kbp). Phylogenetic analysis based on the *B646L* gene sequences (encoding the p72 capsid protein) showed the virus inoculum grouped in the genotype II cluster together with an ASFV Georgia 2007 isolate [13] (Figure S1). A comparison at the p72 nucleotide sequence level revealed that the virus inoculum was 100% identical to previously published sequences of an ASFV isolated in Ghana [48].

### 3.4. Aggregate Oral Fluid Evaluation

#### Virus Inoculation and Clinical Observations

**Figure 3:**
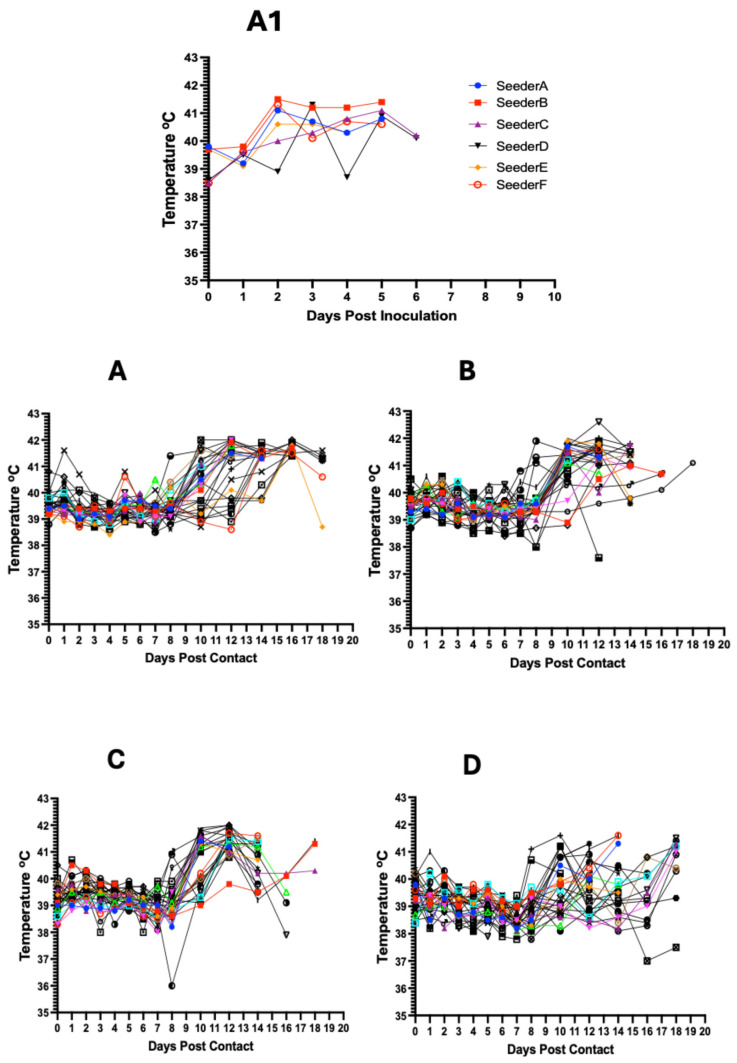

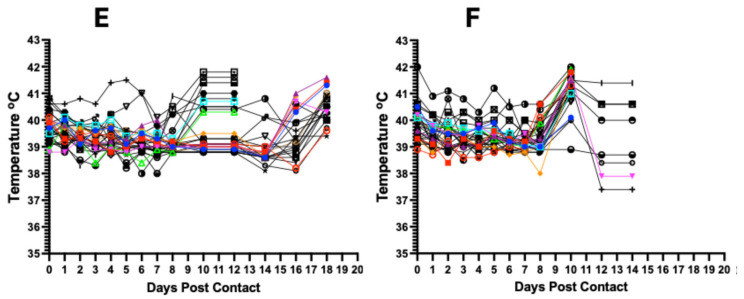
Kinetics of rectal temperature in contact pigs in response to ASFV infection. The overall trend shows increasing rectal temperatures from 10 dpc onwards with the onset of severe clinical symptoms (**A**–**F**); each temperature curve represents response of an individual pig. Temperature response in seeder pigs shows an increase at 2–3 dpi (**A1**).

**Figure 4:**
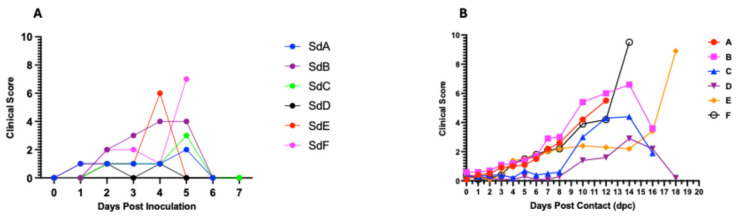
Dynamics of clinical scores in seeder (**A**) and in contact pigs (mean score) for each per pen (**B**). The data shows rising clinical scores over time with the onset of clinical signs and progression of the disease.

Inoculation of the seeder pigs with 0.5 mL of the spleen homogenate resulted in elevated rectal temperature response by 2–3 dpi and onset and development of clinical signs by 2–4 days (Figure 3A1 and Figure 4A). The majority of contact pigs in the six pens exhibited an increase in rectal temperatures, starting at 10–11 dpc, and in several cases, a decrease in rectal temperatures below the normal in pigs exhibiting severe clinical signs or in the terminal stages of disease progression (Figure 3A–F). Clinical observations in contact pigs depicted an onset of rising mean clinical scores at 7–8 dpc in Pen A, B, E, and F and at 10 dpc in Pen C and D (Figure 4B).

### 3.5. ASFV Genomic Detection in Seeder Pigs

**Figure 5:**
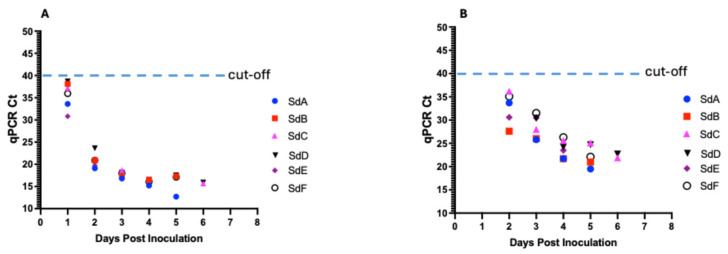
Kinetics of ASFV DNA in seeder pigs in response to intramuscular inoculation with 10% spleen homogenate. Detection of DNA in blood (**A**) and in oropharyngeal swabs (**B**).

Blood samples collected from all six seeder pigs tested positive for ASFV DNA at 1 dpi, with Ct values ranging from 30.82 to 38.66 (Figure 5A, Table 1). Ct values for all seeder pigs declined with the onset of clinical signs and disease progression from the mean Ct value of 35.75 at 1 dpi to 16.4 at 5 dpi and 15.8 at 6 dpi. In contrast, ASFV DNA was detectable in the oropharyngeal swabs at a later timepoint at 2 dpi (Ct value range: 27.6–36.2) (Figure 5B, Table 1). Ct values for all seeder pigs declined temporally with the onset of clinical signs and disease progression from the mean Ct value of 32.64 at 2 dpi to 22.48 at 5 dpi and 22.3 at 6 dpi.

### 3.6. ASFV Genomic Detection in Blood and Oropharyngeal Swabs of Contact Pigs

To determine the infection status and kinetics of ASFV transmission among contact pigs and how these correlated with aggregate oral fluid detections at the pen level (Pen A–F), we tested blood and oropharyngeal swabs of individual pigs from 0 to 18 dpc, the end of the sampling period.

#### 3.6.1. Detection in Pen A

**Figure 6:**
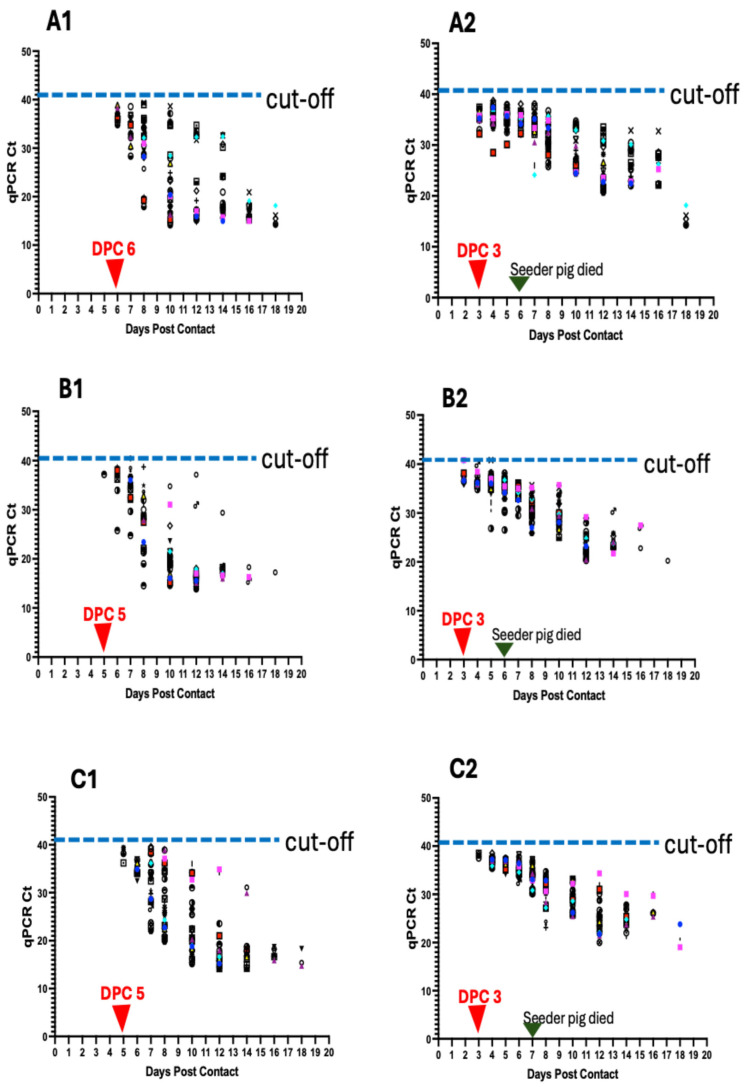

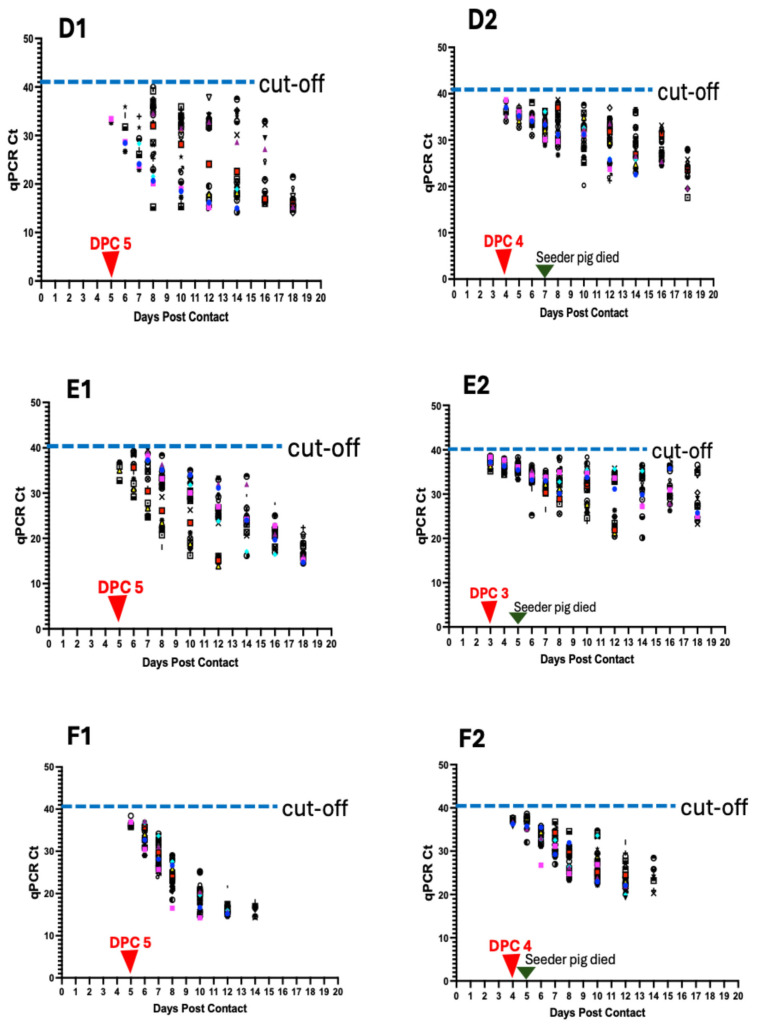
ASFV genomic detection in individual pigs in the six pens (**A**–**F**). Detection of ASFV genomic material in the blood of individual pigs per pen (**A1**–**F1**) and in the oropharyngeal swabs (**A2**–**F2**). Data shows earlier detection of ASFV DNA in oropharyngeal swabs than in blood. The seeder pigs died 5–7 days post-inoculation.

**Figure 7:**
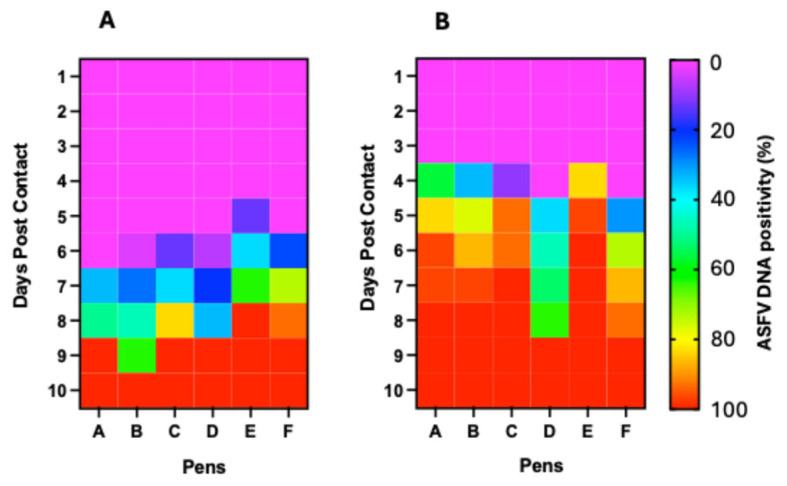
Heat map of ASFV DNA positivity in blood (**A**) and oropharyngeal swabs (**B**). Both figures display a temporal increase in ASFV positivity and also demonstrate earlier detection of ASFV DNA in the oropharyngeal swabs than in blood.

Initial detection of ASFV genomic material in the blood of contact pigs occurred at 6 dpc in 33.3% (10/30) of pigs (Ct value range: 38.32 to 34.84) (Figure 6A1; Table 1). The proportion of pigs that tested positive increased to 50% at 7 dpc (Ct value range: 28.36–38.56) and to 100% at 8 dpc (Ct value range: 39.32 to 17.95) (Figure 7A). There was a decreasing trend in Ct values from 1 dpc to 18 dpc (Figure 6A1). Seeder Pig A died at 6 dpi. For oropharyngeal swabs, initial detection of ASFV genomic material occurred at 3 dpc in 56.7% (17/30) of pigs (Ct value range: 32.21–37.4) (Figure 6A2; Table 1). The proportion of pigs testing positive increased to 96.6% (29/30) at 5 dpc (Ct value range: 30.12–37.91) and 100% at 7 dpc (Ct value range: 24.13–38.19) (Figure 7B). Similarly, there was a decreasing trend in Ct values, as described above (Figure 6A2).

#### 3.6.2. Detection in Pen B

Initial detection of ASFV genomic material in the blood of contact pigs occurred at 5 dpc in a pig indicating 3.3% positivity (Ct value: 37.19) (Figure 6B1; Table 1). The proportion of pigs that tested positive increased to 26.7% (8/30) at 6 dpc (Ct value range: 25.79–39.12) and 100% positivity at 10 dpc (Ct value range: 14.60–34.77) (Figure 7A). There was a decreasing trend in Ct values from 1 dpc to 18 dpc (Figure 6B1). Seeder Pig B died at 6 dpi. For oropharyngeal swabs, initial detection of ASFV genomic material occurred at 3 dpc in 10 pigs (33% positivity) within a Ct value range of 35.95–39.83 (Figure 6B2; Table 1). The proportion of positive animals increased to 76.7 (23/30) at 4 dpc (Ct value range: 34.82–39.92), 86.7% (26/30) at 5 dpc (Ct value range: 26.83–39.83), and 100% at 7 dpc (Ct value range: 29.50–35.51) (Figure 7B). There was a decreasing trend in Ct values over time, as described above (Figure 6B2).

#### 3.6.3. Detection in Pen C

Initial detection of ASFV DNA in the blood of contact pigs occurred at 5 dpc in 4 pigs (13.3% positivity), within a Ct value range of 36.2–39.41 (Figure 6C1; Table 1). Thirty-seven percent (11/30) of the pigs tested positive at 6 dpc (Ct value range: 32.43 to 37), 83.3% (25/30) at 7 dpc (Ct value range: 28.17–39.61), and 100% at 8 dpc (Ct value range: 20.28 to 38.89) (Figure 7A). There was a decreasing trend in Ct values from 1 dpc to 18 dpc (Figure 6C1). Seeder Pig C died at 7 dpi. For oropharyngeal swabs, initial detection of ASFV DNA occurred at 3 dpc in 3 pigs (10% positivity), with Ct values of 38.5, 37.51, and 38.3 (Figure 6C2; Table 1). The proportion of pigs that tested positive increased to 93.3% (28/30) at 4 dpc (Ct value range: 35.25–38.57). There was 100% positivity at 6 dpc (Ct value range: 32.45–38.21) (Figure 7B). Similarly, there was a decreasing trend in Ct values over time (Figure 6C2).

#### 3.6.4. Detection in Pen D

Initial detection of ASFV DNA in the blood of contact pigs occurred at 5 dpc in 2 pigs (6.7% positivity), within a Ct value range of 32.67–33.4 (Figure 6D1; Table 1). The proportion of pigs that tested positive increased at 6 dpc to 20% (6/30) (Ct value range: 32.43–37) and at 7 dpc to 33.3% (10/30) (Ct value range: 26.71–35.96). There was 100% positivity at 8 dpc (Ct value range: 15.24–39.19) (Figure 7A) and a decreasing trend in Ct values from 1 dpc to 18 dpc (Figure 6D1). Seeder Pig D died at 7 dpi. For oropharyngeal swabs, initial detection of ASFV DNA occurred at 4 dpc in 11 pigs (36.7% positivity), within a Ct value range of 34.11–39.04 (Figure 6D2; Table 1). The proportion of pigs that tested positive increased to 46.7% (14/30) at 5 dpc (Ct value range: 34.11–37.95), 53.3% (16/30) at 6 dpc (Ct value range: 30.99–38.10), 63.6% (21/30) at 7 dpc (Ct value range: 28.89–36.22), and 100% positivity at 8 dpc (Ct value range: 32.45–38.21) (Figure 7B). There was a decreasing trend in Ct values, as described above (Figure 6D2).

#### 3.6.5. Detection in Pen E

Initial detection of ASFV DNA in the blood of contact pigs occurred at 5 dpc in 4 pigs (13.3% positivity), within a Ct value range of 32.78–36.78 (Figure 6E1; Table 1). Eleven pigs (36.7%) tested positive at 5 dpc (Ct value range: 29.19–39.22) and 19 pigs (63.3%) at 7 dpc (Ct value range: 24.72–39.46). All the pigs (100%) tested positive at 8 dpc (Ct value range: 18.07–38.32) (Figure 7A). There was a time-dependent decrease in Ct values at 1 dpc through 18 dpc (Figure 6E). Seeder Pig E died at 5 dpi. For oropharyngeal swabs, initial detection of ASFV DNA occurred at 3 dpc in 25 pigs (83.3% positivity), within a Ct value range of 35.19–38.62 (Figure 6E2; Table 1). The proportion of pigs that tested positive increased to 96.7% (29/30) at 4 dpc (Ct value range: 35.21–38.2). There was 100% positivity at 5 dpc (Ct value range: 33.32–38.27) through to 10 dpc (Figure 7B) and a temporal reduction in Ct values from 1 dpc to 18 dpc (Figure 6E2).

#### 3.6.6. Detection in Pen F

Initial detection of ASFV DNA in the blood of contact pigs occurred at 5 dpc in 7 pigs (23.3% positivity), within a Ct value range of 35.56–38.41 (Figure 6F1; Table 1). Twenty-two animals (73.3%) tested positive at 6 dpc (Ct value range: 30.45–37.12) and 28 pigs (93.3%) at 7 dpc (Ct values range: 24.13–33.64). There was 100% positivity at 8 dpc (Ct values range 14.22–27.52) and afterwards (Figure 7A). The Ct values decreased over time from 1 dpc to 14 dpc (Figure 6F1). Seeder Pig F died at 5 dpi. Initial detection of ASFV DNA in the oropharyngeal swabs occurred at 4 dpc in 9 pigs (30% positivity), within a Ct value range of 32.04–35.6 (Figure 6F2; Table 1). The proportion of pigs testing positive increased to 73.3% (22/30) at 5 dpc (Ct value range: 34.11–35.97), 86.7% (26/30) at 6 dpc (Ct value range: 26.74–35.97), and 100% at 8 dpc (Ct value range 24.63–30.89) and afterwards (Figure 7B). There was a temporal reduction in Ct values from 1 dpc to 14 dpc (Figure 6F2).

### 3.7. ASFV Genomic Detection in Aggregate Oral Fluids

**Figure 8:**
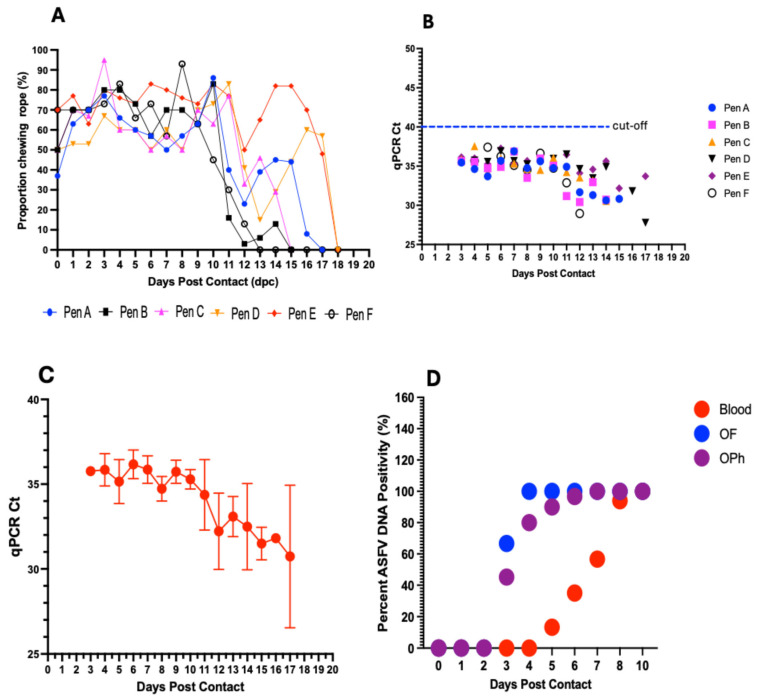
Evaluation of aggregate oral fluids. Dynamics in the proportion of pigs chewing on the rope in the different pens (A–F) at different timepoints postcontact. On average, more than 60% of pigs in each pen chewed on the rope from 1–10 dpc (**A**). ASFV DNA detection in aggregate oral fluids with initial positive detections at 3 dpc in 4 of the 6 pens and in all pens at 4 dpc and afterwards (**B**). Genomic detection demonstrates a decreasing trend in mean Ct values with an increase in viral load (**C**). Comparison of the sensitivity of ASFV DNA detection in whole blood, oropharyngeal (OPh) swabs, and aggregate oral fluids (OFs) (**D**).

To evaluate the sensitivity of detecting ASFV genomic material in aggregate oral fluids, we examined detection performance at an ASF pen prevalence of 3.2%. Across the pens, the average proportion of pigs that chewed on the rope was above 60% (range: 62–79%) from 1 to 10 dpc. From 11 dpc and onwards, the numbers declined progressively with the onset and severity of clinical signs (Figure 8A).

Initial detection of ASFV DNA in aggregate oral fluids occurred at 3 dpc in 4 out of the 6 pens, Pen A (Ct value: 35.46), Pen B (Ct value: 35.63), Pen C (Ct value: 35.85), and Pen E (Ct value: 36.14) (Figure 8B and Table S2). Aggregate oral fluids in all six pens (100% pen detection rate), including Pen D and Pen F, occurred at 4 dpc, within a Ct value range of 34.63–37.55 (Figure 8B and Table S2). Thereafter, ASFV DNA was consistently detected in all pens (A–E) until 14 dpc, after which the pigs became too depressed and lethargic to chew on the rope (Figure 8A and Table S2). Overall, there was a decreasing trend in the average Ct values with disease progression, with mean Ct values decreasing from 35.85 at 4 dpc to 30.74 at 17 dpc (Figure 8C).

Comparing the sensitivity of ASFV DNA detection in aggregate OFs for an infected pen with that in blood and oropharyngeal swabs in individual animals, viral DNA was detected in aggregate OFs as early as 3 dpc (Ct value range: 35.46–36.14) (Figure 8D; Table 1), in contrast to 5 dpc (Ct value range: 32.67–39.41) in blood (Figure 8D; Table 1). Detections in oropharyngeal swabs of some individual pigs coincided with aggregate OF detections, with initial detection in the former sample type also occurring at 3 dpc in the same 4 pens. The remaining 2 pens were positive for both oropharyngeal swabs in some individual pigs and for aggregate OFs at 4 dpc (Figure 8D). Aggregate OFs from 4 out of 6 pens (67%) tested positive at 3 dpc, for a sensitivity of 29 to 90% (with a 95% credible interval), and 100% at 4 dpc (Ct value range: 34.63–37.55), for an estimated sensitivity of 59 to 99.6% (with a 95% credible interval), and thereafter, until 10 dpc (Figure 8D). The initial detection rate in oropharyngeal swabs at 3 dpc was comparatively lower, showing a median pen positivity of 45.2% (range: 10–83.3%), followed by an increase in positivity at subsequent timepoints to 80% (4 dpc; range: 30–93.3%), 90% (5 dpc; range: 46.7–100%), 96.7% (6 dpc; range: 53.3–100%), 100% (7 dpc; range: 73.6–100%), and 100% (8 and 10 dpc) (Figure 8D). For detection in individual blood samples, the earliest timepoint of positive DNA detection was 5 dpc, with a median pen positivity of 13.3% (range: 3.3–23.3%), followed by a gradual increase in positivity at subsequent timepoints to 35% (6 dpc; range: 20–73.3%), 56.7% (7 dpc; range: 33.3–93.3%), 94% (8 dpc; range: 63.3–110%), and 100% (10 dpc) (Figure 8D).

### 3.8. Environmental Sample Testing: Water Nipple and Fecal Swabs

In a preliminary evaluation of environmental samples, water nipples and fecal swabs, to support ASF surveillance, we performed daily pen-level sampling from 0–10 dpc. The qPCR results exhibit variability in detection performance amongst individual pens at different timepoints. For water nipples swabs, there was a positive ASFV DNA detection in Pen E, at 2 dpc (Ct value 36.3). Four pens (A, B, C, and D) out of 6 were positive at 4 dpc (Ct values: 34.4, 35.1, 36.9, and 35.5), a 67% pen detection rate (Table S3). Thereafter, there were inconsistent and sporadic positive DNA detections until 9 dpc (Table S3). The earliest positive ASFV DNA detection in fecal samples occurred at 4 dpc in three pens (A, B, and D) (Ct values: 31.33, 35.88, and 33.88), indicating a 50% pen-level detection rate (Table S3). Thereafter, there were inconsistent and sporadic detections in the various pens until 8 dpc. Four pens (Pen A, D, E, and F) out of 6 tested positive (Ct values: 33.45, 35.75, 34.12, and 32.9), indicating a 67% pen-level detection rate. All six pens (A–F) tested positive (Ct values: 29.89, 35.47, 31.7, 34.04, 34.09, and 31.17) at 10 dpc, a 100% detection rate (Table S4).

### 3.9. Mortality in Contact Pigs

**Figure 9:**
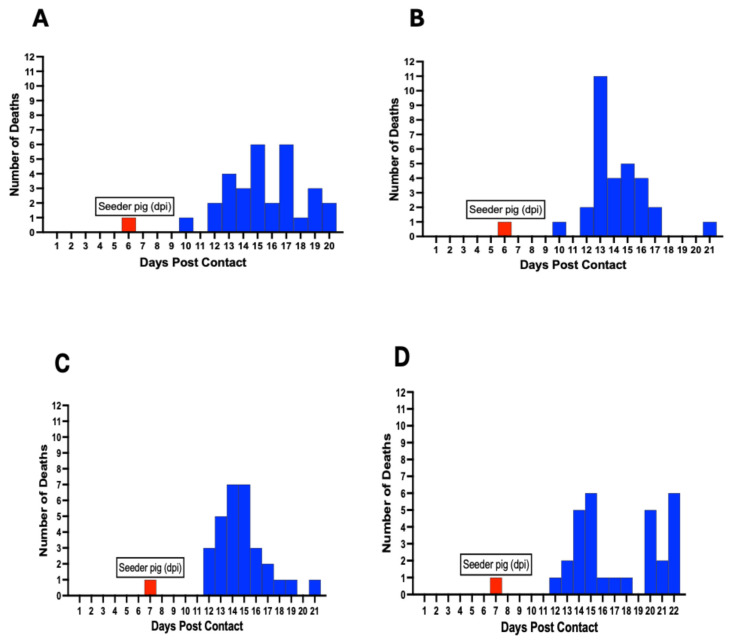

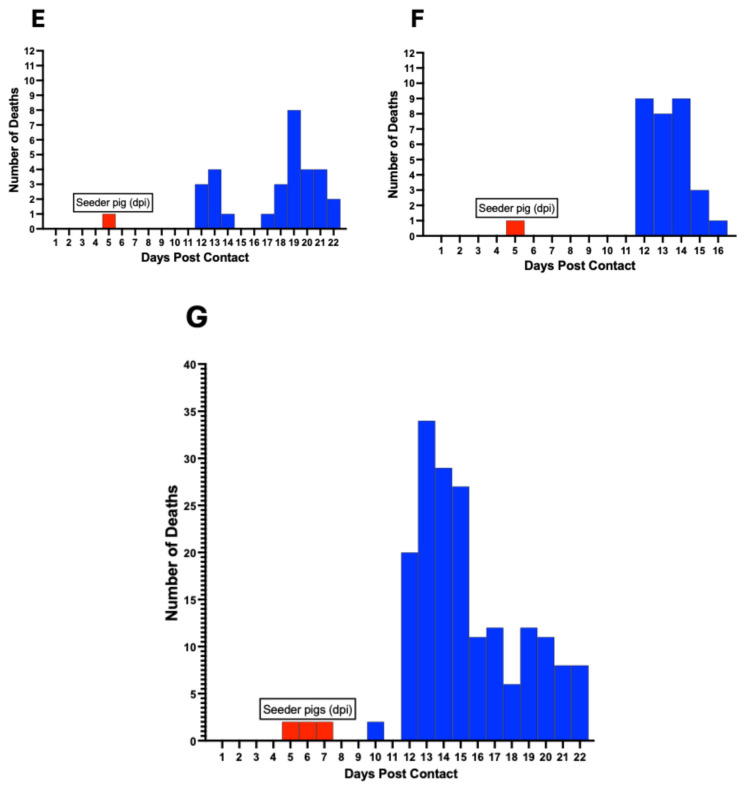
ASF mortality curve in individual pens (**A**–**F**) showing variability in the death rate at different timepoints postcontact. Mortalities occurred between 10–22 dpc. The cumulative mortality curve for all six pens is shown (**G**). Red bars denote day of seeder pig death post-inoculation and introduction in each pen.

Mortalities among contact pigs occurred between 10 and 22 dpc, with the earliest deaths occurring in Pen A and B at 10 dpc (Figure 9). The first deaths in Pen C, D, E, and F occurred at 12 dpc (Figure 9). There was non-uniformity in the mortality curves for the different pens, with Pen F showing the highest number of daily mortalities, occurring between 12 and 16 dpc (Figure 9F). Cumulatively, the highest number of deaths on a single day across all six pens was observed between 12 and 15 dpc, accounting for more than half (61%, 110/180) of the mortalities (Figure 9G).

## 4. Discussion

African swine fever is a highly contagious viral disease of domestic and wild pigs that poses a significant threat to the global swine industry [6,49,50]. There is no effective and widely available vaccine, and, therefore, control and prevention in the US and North America generally are dependent on strong biosecurity measures, movement restrictions, and surveillance for early detection. The current surveillance strategy relies on collection of individual blood samples, the approved sample type for live pigs, for early ASF detection. This approach is laborious and unsustainable in commercial operations with large numbers of pigs. Our study represents the first of its kind that systematically and consistently collected daily aggregate oral fluids as well as individual blood and oropharyngeal swabs from contact pigs across six pen replicates involving 186 pigs to evaluate the use of aggregate oral fluids as an alternative sampling method for ASF surveillance. It also represents the first study that evaluated the sensitivity of the sampling method at a lower ASFV pen prevalence (3.2%) with the exclusion of seeder pigs during aggregate oral fluid sample collection. Unlike previous studies, we performed initial amplification of the virus inoculum in the target host species in phase 1 of the study to confirm virulence, minimize possible attenuating mutations that may result from vitro cell culture propagation, and to ensure the virus retained transmissibility to contact hosts. Additionally, we performed an in vivo pre-study, in phase II, to determine the minimum dose of the virus that induces acute ASF in the target host with survival up to 5–6 days to generate the seeder pigs.

Several studies have been carried out previously that evaluate the utility of aggregate oral fluids for early detection and surveillance of ASFV [12,34,35]. These studies vary in experimental design as well as ASFV pen prevalence, virus phenotype, and sample size, including sampling strategy. In one study, using the moderately virulent ASFV Malta’78 (genotype I) strain [51], a 50% pen prevalence (*n* = 10), and intranasal inoculation of seeder pigs (n = 5), a ASFV genome was detected in the oropharyngeal swabs of the contact pigs at around 4 dpc, regardless of dosage [46]. In other studies, using the ASFV Georgia 2007/1 (genotype II) and ASFV Lisbon 60 (genotype I) strains, administered via the intramuscular route and based on small sizes, with an ASFV pen prevalence of 50–100%, ASFV genomic material was detected in aggregate oral fluids at 2–3 dpi [35,52]. These studies are unlikely to represent the most probable scenario of ASFV introduction into naïve swine herds, whereby 50% of the herd is infected simultaneously, with an equal risk of exposure to ASFV and subsequent early detection via surveillance.

Recently, a study was performed using the highly virulent Georgia 2007/1 strain and the moderately virulent Malta’78 strain. The seeder pigs were inoculated via the intramuscular route and introduced to contact pigs at an ASF pen prevalence of 4–5% (*n* = 20–25 pigs) [34]. In this study, the contact pigs as well as the seeder pigs were allowed to chew on the rope during aggregate oral fluid sample collection. ASFV genomic DNA was detected in the whole blood of seeder pigs at 1–3 dpi in aggregate oral fluids at 3–5 days postcontact, 2–3 days prior to the death of the seeder pigs inoculated with virulent ASFV Georgia 2007/1 and 4–5 days prior to the death of seeder pigs inoculated with ASFV Malta’78 [34].

In our study, we evaluated pen-based aggregate oral fluid detection of ASFV genomic material using a highly virulent ASFV isolate, obtained from a severe ASF outbreak in Ghana. The seeder pigs were inoculated via the intramuscular route and introduced at a lower ASFV pen prevalence of 3.2%, and in contrast to other studies, the seeder pigs did not chew on the rope during aggregate oral fluid sample collection. The seeder pigs developed a fever and exhibited clinical signs at 2–4 dpi. Disease progression was fast, and the pigs succumbed at 5–7 dpi, which is consistent with findings in previous studies of pigs using intramuscular inoculation with highly virulent strains of ASFV isolates [53]. ASFV genomic DNA was detected in all seeder pigs earlier in the blood at 1 dpi and then later at 2 dpi in the oropharyngeal swabs (Figure 5A,B).

ASFV genomic material was detected in the aggregate oral fluids as early as 3 dpc in 4 of 6 pens (29 to 90% with 95% credibility) and in all 6 pens (59 to 99% credibility) at 4 dpc after introduction of the seeder pigs. ASFV was detected in 10 to 83% of the individual pig oropharyngeal swabs the same day as the aggregate OF detection and in 3% to 33% of individual pig blood samples 1–3 days after the aggregate OF detection. These findings suggest that OF sampling is capable of detecting ASFV incursion in a naïve swine herd prior to detection by individual blood tests. Our study shows that there is a 100% chance of detecting ASFV at 4 dpc upon introduction, suggesting that early detection of the virus in a naïve swine herd is possible at the current ASF herd prevalence prior to the onset of apparent clinical signs. We detected ASFV DNA in aggregate OFs at an average of 3 days prior to the death of seeder pigs, which provides a window for early detection of virus incursion. Clinical signs characteristic of ASF became apparent in some contact pigs at 6–7 dpc, which was 3–4 days prior to the occurrence of the first 2 mortality events (Pen A and Pen B, each) and 6 days prior to significant mortalities (Figure 9), presenting an additional opportunity to initiate timely intervention and disease mitigation measures by responding to clinical signs rather than death.

The detection of ASFV genomic material in aggregate oral fluids coincided with the detection of the viral DNA in individual oropharyngeal swabs in all 6 pens and as early as 3–4 dpc (Table 1). In contrast, detection of ASFV DNA in the blood of individual contact pigs occurred, on average, 2 days later (1–3 dpc), showing earliest detection at 5 dpc and a median positivity of 13.3% (Figure 8D). These results suggest that aggregate fluid sampling could be a more sensitive and efficient method for early detection and surveillance of an ASFV incursion, especially in commercial swine operations, compared to testing individual blood samples. The detection of ASFV in the oral fluids and oropharyngeal swabs earlier than in blood is supported by the pathogenesis of ASFV infection via natural transmission. Following ingestion, which represents the natural route of ASFV transmission in domestic pigs [54,55], initial replication of the virus occurs in the tonsils and oropharynx [36,37] and provides a plausible explanation for the early detection of the virus in aggregate fluids and oropharyngeal swabs before detection in blood, supporting the suitability of aggregate oral fluid sampling as an effective tool for surveillance of ASFV. Detection in the blood of seeder pigs prior to detection in oropharyngeal swabs may be explained by the intramuscular administration, which may allow for direct entry of the virus into the circulatory system.

Contact pigs continued to chew on the rope in a significant proportion, with more than 60% of the pigs in every pen chewing until 10 dpc (Figure 8A). This was true even after the appearance of overt clinical signs, likely making it possible to detect the presence of the virus in the oral fluids for an extended period of time as the disease progressed. Although, the detection of ASFV DNA in aggregate oral fluids remained consistent from 4–10 dpc across all pens, it occurred at high Ct values (between 33.5 and 37.4 until 10 dpc), suggesting the presence of low amounts of ASFV genomic material. This may pose a challenge to further virus characterization via whole-genome sequencing. The high Ct values could also be due to environmental contaminants that inhibit PCR sensitivity. Together, these observations suggest the need for research directed at improving the sensitivity (improve Ct values) of aggregate oral fluid detection via improved sample processing and development of enhanced virus capture or enrichment protocols.

The temporal dynamics in the proportion of pigs positive for ASFV genomic material in the blood and oropharyngeal swabs (Figure 7A,B) and the mortality curves (Figure 9) are in keeping with previous studies, demonstrating that ASFV in a swine herd spreads slowly but steadily, taking up to a few weeks from the beginning of an incursion to the appearance of overt disease, requiring time to detect the virus in contact pigs [34,56]. The proportion of pigs positive at the early stages of viral DNA detection varied between pens, which may depend on the hyperactive behavior of the seeder pig and the extent of virus shedding and contamination of the environment. However, we observed the virus spread rapidly through the herd and infected nearly all pigs in all pens by 8–10 dpc. The uneven spread of the virus among the different pens is in keeping with the lack of uniformity in the mortality curves among the pens (Figure 9). The first 2 deaths occurred at 10 dpc, with a significant number of deaths occurring only after 12 days (almost 2 weeks) post-introduction of the seeder pigs, thus supporting the observation about the stealthy nature of initial ASFV incursion.

The lack of a reliable non-invasive sampling method for ASFV detection remains a major constraint for ASFV outbreak surveillance in commercial swine operations in North America. Consequently, we evaluated, as a proof of concept, environmental sampling (fecal and water nipple swabs) to support ASF surveillance. Our results show that although early detection of ASFV genomic material in fecal (at 4 dpc) and water nipple (at 2–3 dpc) swabs at the pen level may be possible, the lack of consistency in detection performance (Tables S3 and S4) could be due primarily to variability in sample collection techniques and indicates the need for further research on optimizing and standardizing the sampling techniques and extraction protocols.

In our study, we generated the seeder pigs via intramuscular inoculation of a highly virulent ASFV. The rapid disease progression in these pigs is in keeping with previous studies [53]. We hypothesize that intramuscular inoculation of the virulent isolate in the current study may have induced significant virus shedding, resulting in widespread environmental contamination. It is also hypothesized that disease progression in the seeder pigs and contact pigs as well as environmental contamination could be moderated when seeder or contact pigs are infected through ingestion via contaminated feed, the natural route of ASFV transmission, or if a low or moderately virulent ASFV strain is used to inoculate the seeder pigs. These scenarios may mimic the likely paths for ASFV incursion and manifestation of disease progression in a commercial swine farm in non-endemic settings. Further studies are required to address these hypotheses.

## 5. Conclusions

In conclusion, we have demonstrated that aggregate oral fluid sampling has the potential to be an effective method for early detection of ASFV incursion in naïve swine herds to support disease surveillance in commercial swine operations in North America and may provide earlier detection than individual animal blood sampling. Detection of ASFV genomic material in aggregate oral fluids from contact pigs occurred as early as 3–4 dpc, 1–3 days earlier than in blood. Detection in aggregate oral fluids coincided with detections in oropharyngeal swabs, suggesting that ASFV may be detected in oral swabs and oral fluids earlier than blood and further supporting the potential utility of aggregate OF sampling for ASF surveillance that could be adopted by the swine industry.

## Figures and Tables

**Table 1:** Summary of mortality and ASFV genomic detection in aggregate oral fluid, blood, and oropharyngeal swabs of seeder and contact pigs.

Pen ID	Seeder Pig Mortality (dpi)	Initial Positive OF Detection (dpc)	Initial Positive Detection in Seeder Pigs (dpi)		Initial Positive Detection in Contact Pigs (dpc)	
			Blood	Oph Swab	Blood	Oph Swab
A	6	3 (35.46)	1 (33.6)	2 (33.7)	6 (34.34–37.4)	3 (35.21–37.10)
B	6	3 (35.63)	1(38.12)	2 (27.6)	5 (37.19)	3 (35.95–39.83)
C	7	3 (35.85)	1(37.21)	2 (36.2)	5 (36.20–39.41)	3 (37.51–38.50)
D	7	4 (35.7)	1 (38.66)	3 (30.4)	5 (32.67–33.40)	4 (34.11–39.04)
E	5	3 (36.14)	1 (30.82)	2 (30.6)	5 (32.78–36.78)	3 (35.19–38.62)
F	5	4 (35.68)	1 (35.94)	2 (35.01)	5 (35.76–38.41)	4 (35.61–37.71)

Note: qPCR Ct values are shown in parenthesis; OF = oral fluid; Oph = oropharyngeal.

## Data Availability

All relevant data is in the paper and its Supporting Information files.

## Supplementary Materials

The following supporting information can be downloaded at: https://www.mdpi.com/article/10.3390/v17081089/s1, Figure S1: Phylogenetic analysis of ASFV isolates based on the *B646L* gene sequences encoding the p72 capsid protein. The tree shows a grouping of the Ghana ASFV virus (purple arrow) inoculum with the p72 genotype II cluster. Table S1: Clinical signs and scoring criteria for ASF in experimental pigs; Table S2: ASFV qPCR Ct values of DNA detections in aggregate oral fluids from contact animals in pens at different timepoints post-contact; Table S3: ASFV qPCR Ct values of DNA detections in water nipple swabs from the pens at different timepoints post-contact/dpi; Table S4: ASFV qPCR Ct values of DNA detections in fecal swabs from the pens at different timepoints post-contact/dpi.
