# Supplementary material for: Evaluation of Aggregate Oral Fluid Sampling for Early Detection of African Swine Fever Virus Infection
Source: Viruses. 2025 Aug 6;17(8):1089. doi: 10.3390/v17081089 (PMC12390537; doi:10.3390/v17081089)
Supplement: Supplementary file 1 [file viruses-17-01089-s001.zip › Supplemental Figure S1.pdf]

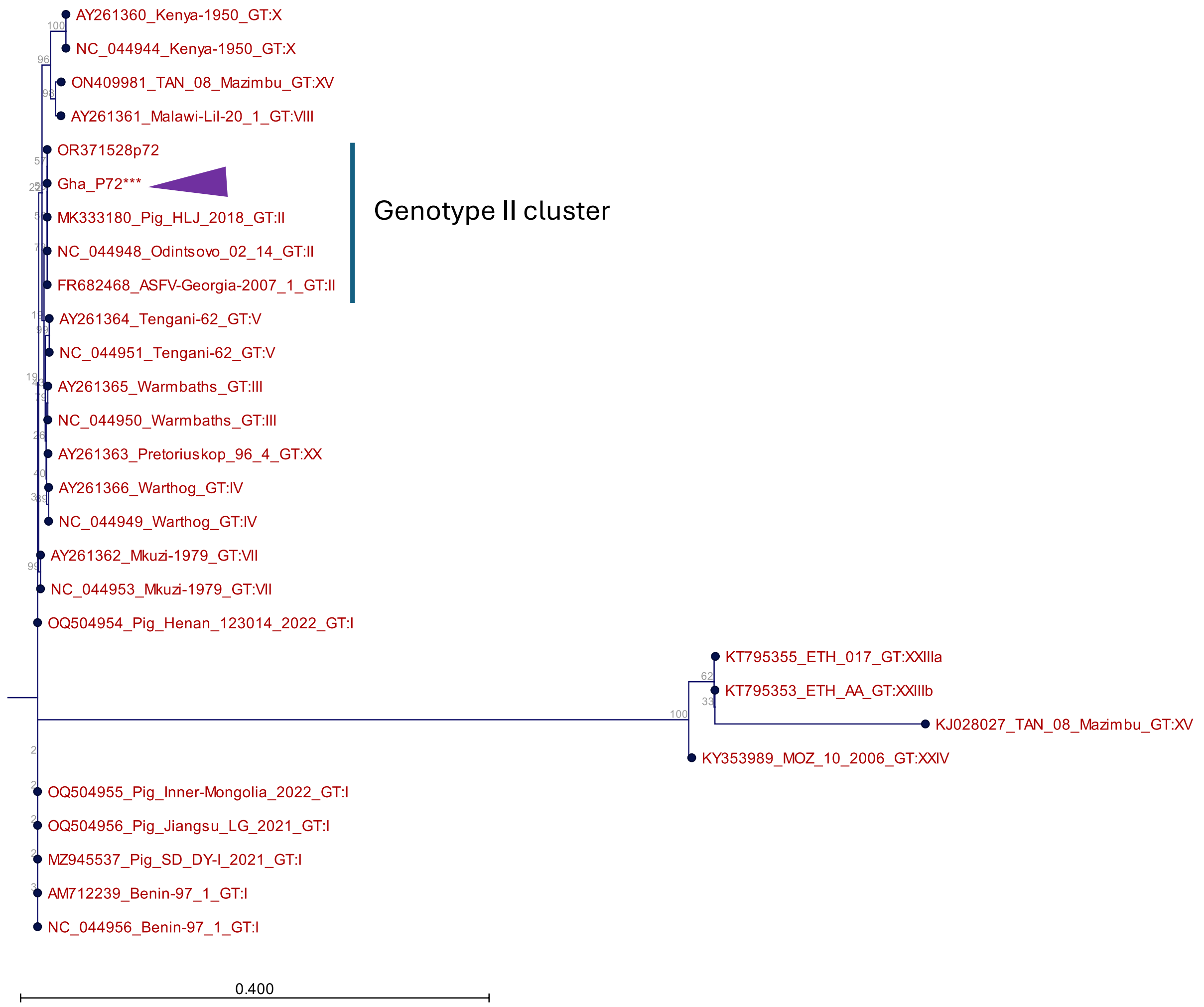

Supplemental Figure S1. Phylogenetic analysis of ASFV isolates based on the *B646L* gene sequences encoding the p72 capsid protein. The tree shows grouping of the Ghana ASFV virus (purple arrow) inoculum with the p72 genotype II cluster.
