# Supplementary material for: Evaluation of Aggregate Oral Fluid Sampling for Early Detection of African Swine Fever Virus Infection
Source: Viruses. 2025 Aug 6;17(8):1089. doi: 10.3390/v17081089 (PMC12390537; doi:10.3390/v17081089)
Supplement: Supplementary file 1 [file viruses-17-01089-s001.zip › Supplemental Table S1.pdf]

Supplemental Table S1. Clinical signs and scoring criteria forASF in experimental pigs.

Characteristic	Score	Description
Behavior and mentation	0	Normal, alert response
	1	Mildly obtunded. Slightly reduced liveliness, stands up unassisted, resists restraint or rectal thermometer
	2	Obtunded. Reluctant to stand but will do so when assisted; decreased resistance to restraint or rectal thermometer
	3	Intermittent ataxia, disorientation, can still stand/walk or will not stand/walk even when assisted, still conscious
	4	Moribund. Non-ambulatory, unconscious/nonresponse
Neurologic signs	0	Normal
	2	Unambiguous neurologic signs (e.g. convulsions, seizures)
Defecation	0	Normal to mildly soft stools
	1	Profuse watery diarrhea with/without hematochezia or melena
	2	Severe to marked hematochezia or melena
Body temperature	0	38-40 ^{O}C
	1	Temperature greater than or equal to 40 ^{O}C at any point of study
	2	Temperature greater than or equal to 40 ^{O}C for at least 2 subsequent days
	3	Temperature greater than or equal to 41 ^{O}C
	4	Temperature less than 38 ^{O}C

Adapted: Howey et al. [42]; de Carvalho Ferreira et al. [46]
