# Supplementary material for: Evaluation of Aggregate Oral Fluid Sampling for Early Detection of African Swine Fever Virus Infection
Source: Viruses. 2025 Aug 6;17(8):1089. doi: 10.3390/v17081089 (PMC12390537; doi:10.3390/v17081089)
Supplement: Supplementary file 1 [file viruses-17-01089-s001.zip › Supplemental Table S2.pdf]

Supplemental Table S2. ASFV qPCR Ct values of DNA detections in aggregate oral fluids from contact animals in pens at different timepoints post-contact (DPC).

	Aggregate OF qPCR Ct Values					
**DPC**	Pen A	Pen B	Pen C	Pen D	Pen E	Pen F
0	-	-	-	-	-	-
1	-	-	-	-	-	-
2	-	-	-	-	-	-
3	**35.46**	**35.63**	**35.85**	-	**36.14**	-
4	34.63	35.54	37.55	**35.7**	36	**35.68**
5	33.7	34.73	34.22	35.55	35.28	37.41
6	35.68	34.9	36.02	36.91	37.25	36.23
7	36.9	36.86	35.33	35.66	35.3	35.1
8	34.76	33.52	34.66	35.22	35.67	34.52
9	35.62	35.98	34.53	35.81	35.81	36.64
10	34.76	35.05	36.06	35.94	35.17	34.75
11	34.92	31.18	34.24	36.5	36.47	32.88
12	31.67	30.42	33.55	34.64	34.1	28.95
13	31.3	32.95	33.18	33.46	34.58	NC
14	30.63	30.73	30.55	34.9	35.63	NC
15	30.82	NC	NC	NC	32.18	NC
16	NC	NC	NC	31.81	NC	NC
17	NC	NC	NC	27.77	33.71	NC

Note: Bold Ct value number indicates initial positive detections in aggregate oral fluids in each pen.
Dash (-): denotes negative PCR result. NC: denotes no chewing on the rope. OF: denotes aggregate oral fluid.
