# Supplementary material for: Evaluation of Aggregate Oral Fluid Sampling for Early Detection of African Swine Fever Virus Infection
Source: Viruses. 2025 Aug 6;17(8):1089. doi: 10.3390/v17081089 (PMC12390537; doi:10.3390/v17081089)
Supplement: Supplementary file 1 [file viruses-17-01089-s001.zip › Supplemental Table S3.pdf]

**Supplemental Table S3.** ASFV qPCR Ct values of DNA detections in water nipple swabs from the pens at different timepoints post-contact/dpi.

| DPC | Water nipples swab qPCR Ct Values | | | | | |
|---|---|---|---|---|---|---|
| | Pen A | Pen B | Pen C | Pen D | Pen E | Pen F |
| 0 | - | - | - | - | - | - |
| 1 | - | - | - | - | - | - |
| 2 | - | - | - | - | **36.3** | - |
| 3 | **34.5** | **35.1** | **36.9** | **35.5** | - | - |
| 4 | 33.6 | 36.6 | - | - | - | - |
| 5 | - | 35.1 | - | - | - | - |
| 6 | 35.8 | 33.9 | - | 36.8 | - | **34.9** |
| 7 | - | - | - | - | - | 35.4 |
| 8 | - | - | - | 36.6 | - | 35.6 |
| 9 | 36.2 | 35.9 | - | - | - | - |
| 10 | 33.2 | - | - | 36.8 | 36 | 36.7 |

**Note:** Bold Ct value number indicates initial positive detections in water nipple swabs in each pen. Dash (-): denotes negative PCR result. DPC: days post contact
