# Supplementary material for: Evaluation of Aggregate Oral Fluid Sampling for Early Detection of African Swine Fever Virus Infection
Source: Viruses. 2025 Aug 6;17(8):1089. doi: 10.3390/v17081089 (PMC12390537; doi:10.3390/v17081089)
Supplement: Supplementary file 1 [file viruses-17-01089-s001.zip › Supplemental Table S4.pdf]

Supplemental Table S4. ASFV qPCR Ct values of DNA detections in fecal swabs from the pens at different timepoints post-contact/dpi.

	Fecal swab qPCR Ct Values					
**DPC**	Pen A	Pen B	Pen C	Pen D	Pen E	Pen F
0	-	-	-	-	-	-
1	-	-	-	-	-	-
2	-	-	-	-	-	-
3	-	-	-	-	-	-
4	**31.33**	**35.88**	-	**33.88**	-	-
5	-	-	-	-	-	-
6	-	-	**33.74**	35.18	-	-
7	-	36.06	-	-	-	**36.65**
8	-	-	-	-	-	-
9	33.45	-	-	35.75.	**34.12**	32.9
10	29.89	35.47	31.7.	34.07.	34.09	31.17

Note: Bold Ct value number indicates initial positive detections in fecal swabs in each pen. Dash (-): denotes negative PCR result. DPC: days post contact
